# 627. CURE ID as a Tool for Curating and Analyzing Drugs Used in COVID-19 Clinical Trials

**DOI:** 10.1093/ofid/ofab466.825

**Published:** 2021-12-04

**Authors:** Reema Charles, Parvesh Paul, Heather Stone, Serghei Gorobet, Belinda Nhundu, Katarzyna M Borkowski, Mili Duggal, Raghavendra Tirupathi

**Affiliations:** 1 Critical Path Institute, Silver Spring, MD; 2 NCATS - NIH, Silver Spring, MD; 3 FDA, Silver Spring, MD; 4 NCATS/NIH, Rockville, MD; 5 Food and Drug Administration, North Bethesda, MD; 6 WellSpan Health, Chambersburg, PA

## Abstract

**Background:**

CURE ID is an internet-based data repository (https://cure.ncats.io/explore) developed collaboratively by FDA and NCATS/NIH. It is designed to capture real-world clinical outcome data to advance drug repurposing and to inform future clinical trials for infectious diseases with high unmet medical need. It also serves as a repository of clinical trials automatically pulled from https://www.clinicaltrials.gov into the CURE ID platform, where they were then manually curated, with the intention of keeping the infectious diseases community updated on the various clinical trials underway. The current study is a descriptive analysis of various therapeutics in clinical trials against COVID-19 on the CURE ID platform.

**Methods:**

Using clinicaltrials.gov we selected those trials addressing therapeutics for COVID-19 and reviewed the drugs used, the current status of the trials, and the phases of development.

**Results:**

As of May 2021, we identified 2,154 clinical trials and 933 drugs from clinicaltrials.gov that met the inclusion criteria. Hydroxychloroquine (n=251) was the most commonly investigated agent, followed by convalescent plasma (n=147), azithromycin (n=98), ivermectin (n=68), mesenchymal Stem Cells (n=63), tocilizumab (n=58), remdesivir (n=53) and favipiravir (n=51). At the time of our analysis, the majority (45%) of the clinical trials were in the recruiting phase, 12% were in the active phase, and 13% of the studies were completed. The majority (31%) of trials were in phase two, followed by phase three (21%) and phase one (10%). The vast majority of the agents were repurposed (92%), while only 8% of the agents were new molecular entities. Remdesivir was the only drug approved for marketing for treatment of certain patients with COVID-19 at the time of our analysis.

**Conclusion:**

Several repurposed and novel drugs are being investigated to treat COVID-19 in clinical trials. CURE ID provides a broad view of the various drugs being researched and serves to keep the scientific community informed. Such a platform may help prevent duplication of efforts and help the scientific community with more coordinated research efforts and larger platform trials that can robustly answer scientific questions during a pandemic.

**Disclosures:**

**All Authors**: No reported disclosures

